# A cholesterol-binding domain in STIM1 modulates STIM1-Orai1 physical and functional interactions

**DOI:** 10.1038/srep29634

**Published:** 2016-07-27

**Authors:** Jonathan Pacheco, Laura Dominguez, A. Bohórquez-Hernández, Alexander Asanov, Luis Vaca

**Affiliations:** 1 Instituto de Fisiología Celular, Universidad Nacional Autónoma de México, Ciudad Universitaria, México, DF 04510, México; 2 Departamento de Fisicoquímica, Facultad de Química, Universidad Nacional Autónoma de México, Ciudad Universitaria, México DF 04510, México; 3 TIRFLabs Inc. 106 Grendon Place. Cary, NC 27519, USA.

## Abstract

STIM1 and Orai1 are the main components of a widely conserved Calcium influx pathway known as store-operated calcium entry (SOCE). STIM1 is a calcium sensor, which oligomerizes and activates Orai channels when calcium levels drop inside the endoplasmic reticulum (ER). The series of molecular rearrangements that STIM1 undergoes until final activation of Orai1 require the direct exposure of the STIM1 domain known as SOAR (Stim Orai Activating Region). In addition to these complex molecular rearrangements, other constituents like lipids at the plasma membrane, play critical roles orchestrating SOCE. PI(4,5)P_2_ and enriched cholesterol microdomains have been shown as important signaling platforms that recruit the SOCE machinery in steps previous to Orai1 activation. However, little is known about the molecular role of cholesterol once SOCE is activated. In this study we provide clear evidence that STIM1 has a cholesterol-binding domain located inside the SOAR region and modulates Orai1 channels. We demonstrate a functional association of STIM1 and SOAR to cholesterol, indicating a close proximity of SOAR to the inner layer of the plasma membrane. In contrast, the depletion of cholesterol induces the SOAR detachment from the plasma membrane and enhances its association to Orai1. These results are recapitulated with full length STIM1.

STIM1 and Orai1 are essential molecular components of the Store Operated Calcium Entry (SOCE), a well-conserved mechanism of Calcium signaling present from insects to humans and critical during the activation of T-cells in the immune response^1^. Orai1 functions as the pore-forming channel activated when intracellular calcium stores are depleted, most notably the endoplasmic reticulum (ER). Under these conditions STIM1 senses the reduction in luminal calcium concentration and undergoes a series of molecular rearrangements that culminate with the exposure of the so-called SOAR (Stim Orai Activating Region) or CAD (CRAC Activation Domain)^2^,^3^ responsible to directly interact and activate Orai channels. The SOAR region covers from amino acids 340 to 450 of STIM1 and falls within the coiled-coil CC2 and CC3 domains^4^. Expression of SOAR has been shown to be sufficient to induce constitutive activation of Orai channels^2^,^3^.

In order for SOAR to interact with the Orai1 N and C-terminal domains^5^, STIM1 must adopt an extended conformation^6^ and migrate to ER regions that are in closed appositions to the plasma membrane (PM). Such regions are recognized as ER-PM junctions^7^,^8^. The molecular mechanism controlling the translocation of STIM1 to ER-PM junctions are not well understood. However, several proteins have emerged recently as partners in facilitating and tethering ER-PM membranes to favor STIM1 movement to these sites^9^. These proteins include septins^10^, synaptotagmins^11^, and the transmembrane protein TMEM110 (also called STIMATE)^12^,^13^. The dynamic formation of these cellular structures seems to be highly complex due to the fact that 70 different proteins participate in this event^12^.

An additional level of complexity arises from the intervention of specific lipids at these microdomains, most notably the PI(4,5)P_2_ that recruits tethered proteins, including STIM1 by mean of a direct association to the lysine-rich region at its C-terminal domain^14^. On the other hand, cholesterol is enriched at ER-PM junctions where oxysterol–binding proteins (OSBP) perform non-vesicular sterol lipid transfer between membranes^9^,^15^. In addition, cholesterol has been shown to be ubiquitous component of specialized PM microdomains^16^,^17^. These participating as signaling platforms to recruit SOCE components at ER-PM junctions^18^,^19^,^20^,^21^. In that regard, disruption of cholesterol microdomains by reducing PM cholesterol via methyl β-cyclodextrin (MβCD) results in the attenuation of SOCE and reduction of the Orai1-STIM1 interaction^20^,^22^. However, the molecular mechanism by which cholesterol modulates STIM1 is not fully understood, most of the evidence points to the inhibition of clustering of STIM1 at cell periphery under reduced cholesterol conditions^18^. In contrast, the modulation of cholesterol on the already formed STIM1-Orai1 complex remains unexplored, in spite of the importance of the lipid’s environment on the regulation of a variety of ionic channels^23^. Here, we investigated the role of cholesterol once the STIM1/Orai1 complex has been formed. All the studies published to this date indicate that cholesterol reduction prior to ER depletion results in the reduction of SOCE, diminishes STIM1/Orai1 association and generates STIM1 puncta at ER regions away from the PM.

Our results show that cholesterol depletion from the PM after the STIM1/Orai1 complex has been assembled results in enhanced SOCE and increased association between STIM1 and Orai1. Furthermore, we show that the mechanism by which cholesterol downregulates Orai1 involves STIM1 association to the PM cholesterol via a novel cholesterol-binding domain (CB domain) contained within SOAR. Most strikingly, a single mutation of isoleucine (I364) disrupts SOAR-cholesterol association and enhances the association of the SOAR domain with Orai1 channels. Similar results are obtained by removing cholesterol from the PM with MβCD or Filipin with SOAR wild type. The SOAR I364A mutant recapitulates the phenotype obtained with wild type SOAR in cells depleted of cholesterol. The effect of reducing cholesterol prior to STIM1-Orai complex formation is not altered in the I364 mutant, indicating that such effect is controlled by a different (yet unidentified) cholesterol-binding domain within STIM1 or its auxiliary proteins. Our experimental results and molecular dynamics simulations strongly suggest that the interaction of SOAR to PM cholesterol provides an anchoring platform for STIM1 to be attached to the PM. These results unveil a detailed molecular mechanism of how STIM1 is directly regulated by cholesterol content at the PM, facilitating or impeding the presentation of the SOAR region to Orai channels. Altogether, our data highlights the importance of enriched cholesterol microdomains to modulate the STIM1-Orai1 interaction after SOCE activation and differentiates this mechanism from the cholesterol actions previous to STIM1/Orai1 complex formation.

## Results

### Role of cholesterol in SOAR-Orai1 interaction

**Figure 1:**
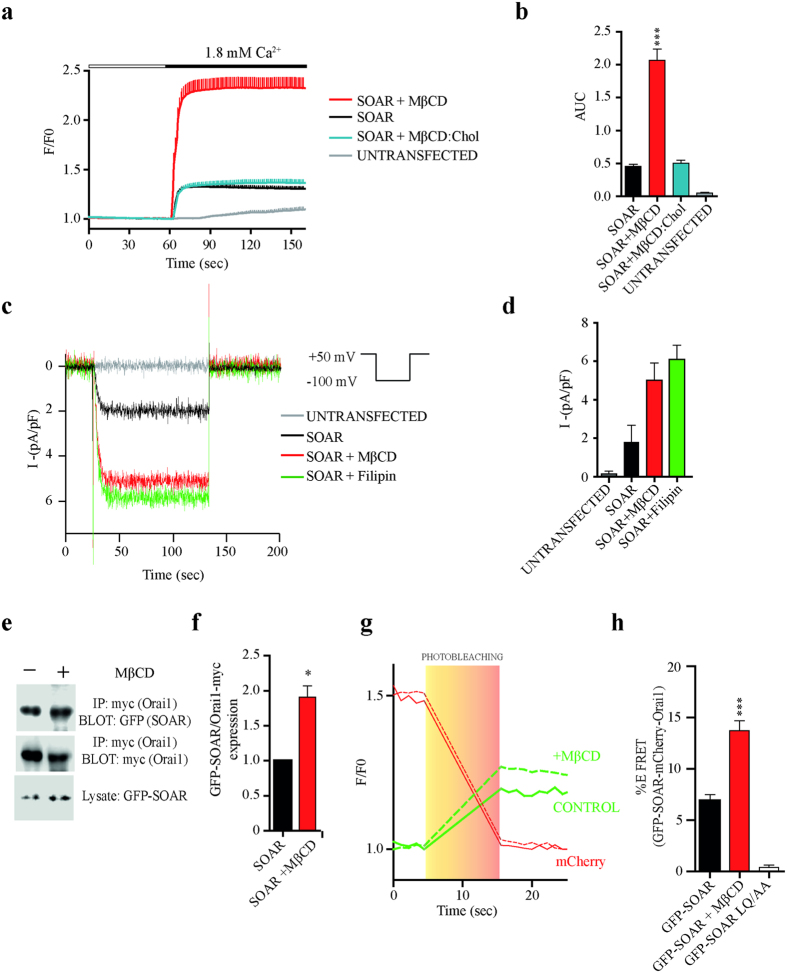
Cholesterol depletion increases functional association of SOAR and Orai1. (**a)** Average calcium response of cells expressing SOAR and Orai1. Control cells present standard cholesterol conditions (Black line), cells depleted of cholesterol with 7.5 mM of MβCD (Red line), cells treated with MβCD:cholesterol (1:1 mole ratio) (Blue line) and untrasfected cells (gray line). For clarity error bars are show only from above of the mean. (**b**) Summary graph bars of area under de curve (AUC) from the calcium addition of control (n = 49 cells), MβCD treated cells (n = 28), MβCD:cholesterol (n = 25) and untransfected cells (n = 11). (**c**) Whole-cell patch-clamp recordings of cells transfected with mCherry-Orai1 and GFP-SOAR. Black trace represent cells expressing SOAR and Orai1 with standard cholesterol conditions, red and green line show cells treated with MβCD and filipin respectively. Gray trace represent cells without transfection. The protocol to obtain these traces is shown at top right. (**d)** Bar graph summarizes current density measured at -100 mV. Data represent mean ± standard deviation from at least 15 cells from 3 transfections. (**e)** Representative blots from at least 4 independent experiments. Control and MβCD treated cells were transfected with Orai1-myc and GFP-SOAR and immunoprecipitated with anti-myc antibody and probe for co-immunoprecipitation of SOAR with anti-GFP antibody. (**f)** Graph bars obtained by densitometry analysis of western blot data. Ratio of co-immunoprecipitated (SOAR) and immunoprecipitated (Orai1) signal of individual experiments were normalized at 1 for control (Black bar) and compared with MβCD treatment (Red Bar). (**g)** Example of acceptor photobleaching FRET experiment. Continuous and discontinuous red lines (acceptor) represent fluorescence of mCherry-Orai1 in control and MβCD treated cells respectively. Continuous and discontinuous green lines show fluorescence of GFP-SOAR with normal cholesterol and poor cholesterol conditions respectively. Light box indicates the photobleaching time-lapse of mCherry. (**h)** Bar graphs summarize FRET efficiencies from control (Black bar n = 136), MβCD treated cells (Red bar n = 90) and the mutant SOAR LQ/AA used as negative control (White bar n = 33). Bars represent mean ± s.e.m. *p < 0.05 and by two-tailed Student’s t-test in f. ***p < 0.0001 by one-way ANOVA with Bonferroni post-test analysis for b and h.

It has been previously demonstrated that the expression of the SOAR domain results in a constitutive interaction with Orai1 channels, activating Orai1 independently of endoplasmic reticulum (ER) calcium store content^2^,^3^. To evaluate the role of cholesterol once the STIM1-Orai (SOCE) complex is established, we used cells overexpressing SOAR and Orai1. By using the SOAR fragment we discarded the effects of inhibition of STIM1 puncta when cholesterol is removed from the PM, a phenomenon that has been previously reported^14^,^18^,^22^. Intracellular calcium measurements were performed in cells treated with methyl-β-cyclodextrin (MβCD). Figure 1a shows cytoplasmic calcium influx mediated by cells expression of SOAR and Orai1. Very interestingly, cells depleted of cholesterol showed a drastic increase of cytoplasmic calcium when compared to cells expressing SOAR and Orai1 with normal cholesterol levels (Fig. 1a). In order to show that this response was dependent on cholesterol levels and not by an unrelated effect of MβCD treatment, we incubated cells with equimolar concentrations of MβCD and cholesterol, producing very similar calcium increments to those obtained in cells expressing SOAR with normal cholesterol levels (Fig. 1a). The addition of extracellular calcium resulted in five times larger calcium influx in cholesterol depleted conditions compared to cells with normal cholesterol levels (Fig. 1b). In addition, treatment with filipin, a cholesterol-binding agent, produced indistinguishable results to those obtained using MβCD (see Supplementary Fig. S1A). Furthermore, calcium entry post-TG addition was also higher in cells expressing SOAR with reduced cholesterol content (see Supplementary Fig. S1C,D).

Correspondingly, we recorded Orai1 currents activated by SOAR upon treatment with MβCD or filipin (Fig. 1c). Resulting in larger currents when PM cholesterol was reduced with either of the 2 agents (Fig. 1d). In contrast, when cholesterol was reduced before the formation of the STIM1-Orai1 complex (using cells expressing SOAR, or full length STIM1 without ER depletion) we observed the already reported effect of reduced calcium entry (see Supplementary Fig. S2). Quantification of cholesterol content with MβCD treatment resulted in a reduction of 84% without affecting significantly cell viability (see Supplementary Fig. S3).

To evaluate if cholesterol was affecting the association between SOAR and Orai1 (one of several feasible mechanisms to explain the enhanced calcium influx), we co-immunoprecipitated SOAR associated to Orai1. The co-immunoprecipitation assay showed an enhanced association of SOAR to Orai1 in cholesterol depleted cells (Fig. 1e). Average densitometric quantification of SOAR co-immunoprecipitated with Orai1 is shown in Fig. 1f. To corroborate this result, we performed acceptor photobleaching FRET studies in cells overexpressing GFP-SOAR and mCherry-Orai1. Figure 1g shows representative fluorescence measurements before and after triggering a photobleaching pulse for mCherry (acceptor). The FRET signal was observed as an increment in GFP fluorescence (donor) after photobleaching the acceptor. Under normal cholesterol levels the FRET efficiency of SOAR-Orai1 was significantly higher than the negative control SOAR LQ/AA (6.86 ± 0.60 FRET efficiency and 0.39 ± 0.20 n = 33 respectively), a SOAR mutant previously reported not to interact with Orai1^2^. However, after removing cholesterol, the FRET efficiency between SOAR-Orai1 was significantly enhanced (13.71 ± 0.97 n = 90). Results are summarized as FRET efficiency in Fig. 1h. These data indicate an increased association of the SOAR-Orai1 complex in cholesterol poor conditions, which may explained the resulting increment in calcium influx.

### A cholesterol-binding domain in SOAR

**Figure 2:**
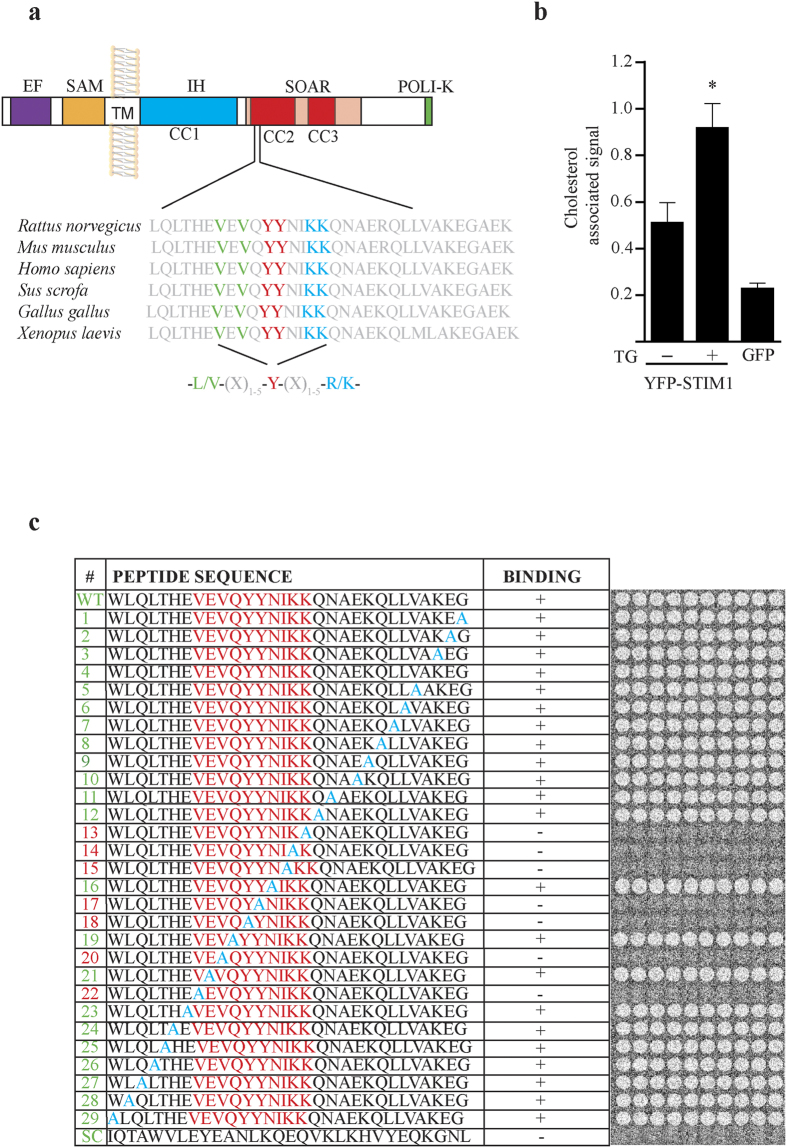
STIM1 interacts with cholesterol in calcium store dependent manner. (**a)** Putative CB domain within SOAR. Topology and sequence alignment of STIM1 show a conserved putative CB domain, this region falls in the first residues of SOAR in CC2. (**b**) Endogenous cholesterol associated to immunoprecipitates of STIM1.Bar graph show cholesterol signal normalized with YFP fluorescence in each immunoprecipitate. Data summarize results from at least 5 experiments. As control GFP was expressed alone and probed to measure the amount of associated cholesterol. (**c**) Peptide array covering from 350 to 380 amino acids of STIM1 and incubated with 10 μM of DHE. Signal was detected by TIRF excitation to eliminate background noise. First line (WT) shows wild type sequence, from line numbered 1 to 29 alanine-scanning mutations were performed, last line (SC) is a scramble sequence as negative control. Red letters depict the putative CB domain. Bars represent mean ± s.e.m. *p < 0.05 by one-way ANOVA with Bonferroni post-test analysis.

The previous section showed that activated SOAR-Orai1 complex is enhanced by cholesterol removal from the PM. In consequence we hypothesized that STIM1 and in particular the SOAR domain may have a cholesterol-binding domain. By analyzing the sequence of STIM1 we identified a putative cholesterol-binding (CB) domain^24^ (Fig. 2a). CB domain of this type are characterized by the consensus sequence L/V-X(1–5)-Y-X(1–5)-R/K, where X represents from 1 to 5 any amino acids before the next conserved residue^24^. Using a method developed by Zheng^25^, we measured the amount of endogenous cholesterol associated to STIM1. The assay consisted in detecting cholesterol incorporated to immunoprecipitates of STIM1 fused to YFP. Following this procedure, we found a constitutive amount of cholesterol associated to STIM1 in conditions where the ER is filled (0.51 ± 0.08 cholesterol associated signal). In comparison, cells treated with TG to deplete the ER showed a greater association of STIM1 to cholesterol (0.97 ± 0.02), which indicates that STIM1 is immunoprecipitated with significant larger amounts of endogenous cholesterol after store depletion, compared to basal conditions (Fig. 2b). GFP was expressed alone to evaluate constitutive association of cholesterol to this tag (Fig. 2b).

To confirm the specific STIM1 interaction with cholesterol we developed specifically for this study a peptide (30 amino acids in length) screening method consisting in a library array comprising from amino acids 350 to 380 of STIM1. Peptide arrays contain almost the entire CC2 domain of STIM1 but excludes the basic patch (382–387), which is the region reported to interact with Orai1^26^,^27^. In addition, the basic patch was discarded from the array to avoid the possibility that the polar head of cholesterol may interact with the peptides mainly through electrostatic interactions^28^. The peptide array was incubated with Dehydroergosterol (DHE), a fluorescent analog structurally and functionally similar to cholesterol^29^. In Fig. 2c the peptide array (WT) show positive binding, meanwhile the last line (SC), show a negative control produced by scrambling the WT sequence, which does not bind DHE. By using alanine substitutions we identified important residues involved in the DHE interaction. Alanine scanning resulted in the abolition of DHE binding when the mutation fell in V357, V359, Y361, Y362, I364, K365 and K366 (Fig. 2c). These data suggest that SOAR presents a CB domain and single mutations on residues constituting the CB domain completely abolished DHE binding *in vitro*.

### Identification of the residues implicated in cholesterol interaction

**Figure 3:**
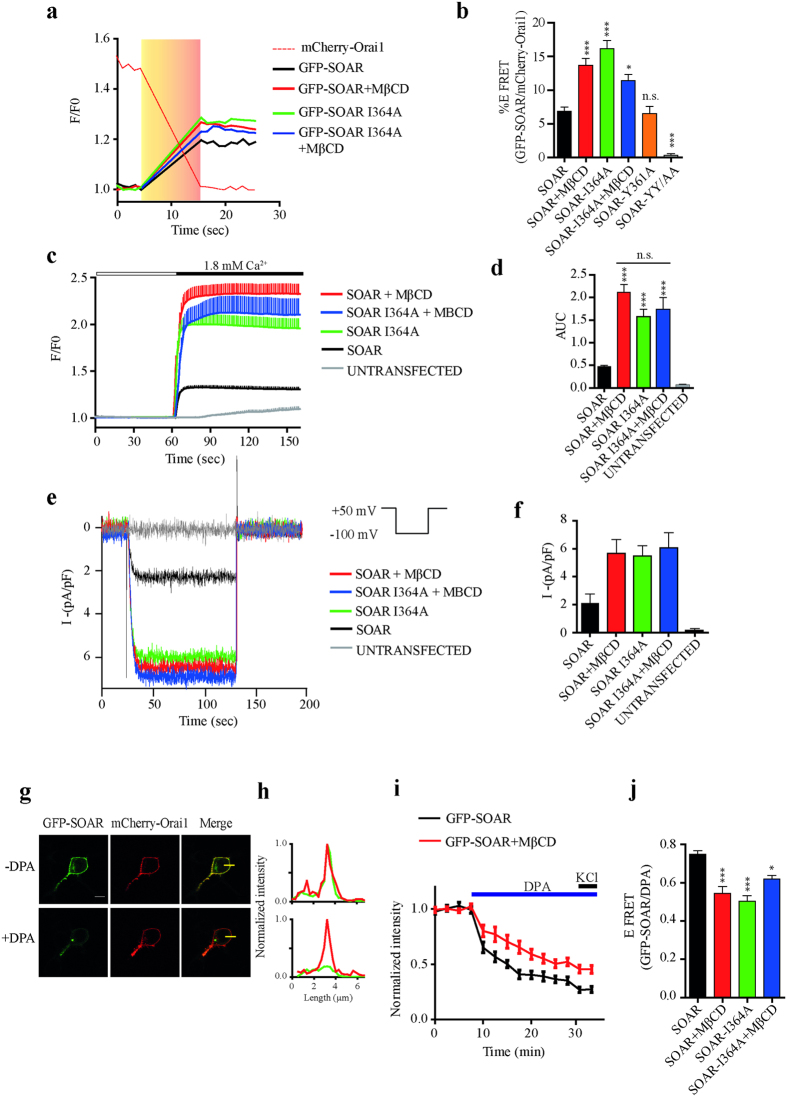
Cholesterol depletion enhances interaction with Orai1 but increases distance with the PM. (**a**) Representative FRET experiments. Continuous black and red lines show fluorescence of GFP-SOAR without and with MβCD respectively. Green and blue lines show fluorescence of GFP-SOAR I364A without and with MβCD respectively. (**b**) Bar graphs summarize FRET efficiencies from SOAR (Black bar n = 136), SOAR plus MβCD (Red bar n = 90), SOAR I364A (Green bar n = 34), SOAR I364A plus MβCD (Blue bar n = 27), SOAR Y361A (Orange bar n = 42) and SOAR YY361/362AA (Gray bar n = 35). (**c**) Average cytosolic calcium measurements of cells expressing SOAR and Orai1 in the absence (n = 49) and presence of MβCD (n = 28) (Black and red trace respectively) and SOAR I364A without (n = 40) and with MβCD (n = 11) (Green and blue trace respectively). For clarity, error bars are shown only from above of the mean. (**d**) Quantification of the area under the curve of experiments in panel **c**. (**e**) Whole-cell patch-clamp recordings of cells transfected with mCherry-Orai1 and GFP-SOAR I364A and wild type. Black and red traces represent cells expressing SOAR and Orai1 with standard and poor cholesterol conditions respectively. Cells expressing Orai1 and SOAR I364A were untreated and treated with MβCD, green and blue line respectively. (**f**) Bar graph summarizes current density from at least 15 cells from 3 independent transfections. (**g**) Example of GFP fluorescence before and after 20 μM of DPA. (**h**) Fluorescence distribution of mCherry-Orai1 and GFP-SOAR obtained by images in g. Upper and lower graph shows before and after DPA addition respectively. (**i**) Time course of GFP quenching after DPA addition. Last two points show the stimulation with high potassium solution. Black line shows GFP-SOAR and red line GFP-SOAR with MβCD. Both were cotransfected with mCherry-Orai1. (**j**) Bar graphs show FRET efficiencies measured after addition of high potassium solution in experiments like shown in panel **i**. SOAR (Black bar n = 16), SOAR plus MβCD (Red bar n = 24), SOAR I364A (Green bar n = 24) and SOAR I364A plus MβCD (Blue bar n = 22). Bars represent mean ± s.e.m. *p < 0.05, ***p < 0.0001 by one-way ANOVA with Bonferroni post-test analysis comparing with SOAR as control. n.s. not significant.

According to the peptide array, residues covering from V357 to K366 form a potential cholesterol interacting site in STIM1. However, mutations of some of these residues might result in a complete disruption of the α-helix or the coiled coil domains leading to the loss of dimer conformation in STIM1 and its subsequent loss of interaction with Orai1, as previously reported^4^,^30^. Such is the case of Y362 involved in the α-helix interactions that controls STIM1 homodimers^4^,^30^. Thus, we searched for residues implicated in cholesterol interactions that do not disrupt the SOAR structure. First, several SOAR mutations covering the potential site for interaction with cholesterol were evaluated to determine if that mutation disrupted the interaction with Orai1 (Fig. 3a). FRET results show that mutations of tyrosine (361 and 362) produced an impaired SOAR-Orai1 coupling, characterized by the loss of FRET signal. Evaluating Y361 resulted in the same levels of FRET efficiency than the control (Fig. 3b) remarking the role of Y362 in the homodimer conformation of SOAR. Meanwhile, testing SOAR I364A produced high levels of FRET efficiency (16.18 ± 1.184% efficiency of FRET) with comparable levels of FRET obtained after MβCD treatment (13.71 ± 0.972% Efret). Removing cholesterol with SOAR I364A produced no significant reduction on FRET efficiency (11.72 ± 0.885 EFret) compared with SOAR I364A with normal cholesterol levels. In this way SOAR I364A in both conditions showed comparable levels of FRET to those obtained with cells expressing SOAR WT with MβCD treatment (13.71 ± 0.972% Efret) (Fig. 3b). All these results indicated that I364 is involved in cholesterol recognition and the mutation I364A did not alter SOAR-Orai1 interactions.

Measurements of calcium influx were conducted with SOAR I364A co-expressed with Orai1. Figure 3c shows calcium increments of SOAR and Orai1 expressing cells with and without MβCD treatment. Interestingly SOAR I364A with and without MβCD presented elevated calcium responses (Fig. 3c). This result further strengthened the idea that I364 participated in cholesterol recognition by SOAR. The area under the curve from experiments in Fig. 3c shows no significant differences between SOAR with MβCD and SOAR I364A with and without MβCD (Fig. 3d). SOAR I364A treated with filipin showed same responses to calcium entry that SOAR I364A untreated (see Supplementary Fig. S4). Electrophysiological recordings confirmed increased Orai1 currents when SOAR I364A was coexpressed (Fig. 3e). Producing same levels of current density than SOAR WT with MβCD (Fig. 3f).

Our results suggest that SOAR must be positioned in closed proximity to the PM in order to interact with plasmalemmal cholesterol. To evaluate if SOAR is located at a suitable distance from the PM, we used FRET between GFP and dipicrylamine (DPA)^31^, a lipophilic nonfluorescent anion that inserts into the hydrophobic core of the lipid bilayer and absorbs emission of green and blue fluorescent proteins^31^. Cells overexpressing both GFP-SOAR and mCherry-Orai1 showed quenching of GFP fluorescence upon DPA addition (Fig. 3g,h). DPA is also used as a voltage sensor that responds to changes in membrane potential^31^. Here, maximum FRET was reached when a high potassium solution was added to the extracellular media, producing the movement of DPA molecules to the inner leaf of the PM, which results in increased FRET with GFP-SOAR (0.74 ± 0.01 FRET efficiency, EFret) (Fig. 3i). In addition, removal of cholesterol resulted in a reduction of FRET efficiency (0.54 ± 0.03 EFret), which indicates a greater distance between SOAR and the PM interface (Fig. 3i). Remarkably, SOAR I364A showed similar FRET efficiency to SOAR WT with MβCD (0.50 ± 0.02 EFret) and cholesterol depletion in this mutant produced only a slightly increase in FRET efficiency (0.62 ± 0.01 EFret) (Fig. 3j).

From the FRET efficiencies we calculated distances between SOAR and the PM, resulting in 3.12 ± 0.075 nm (n = 20) for GFP-SOAR and 3.61 ± 0.097 nm (n = 24) for GFP-SOAR without cholesterol. These results indicate that SOAR distance itself from the PM about 0.5 nm when cholesterol is removed (even tough SOAR remains associated to Orai1). Furthermore, the interaction of SOAR with Orai1 improved when cholesterol is reduced (Fig. 3a,b) indicating that the SOAR distance to the PM is controlled by cholesterol and that it is inversely proportional to its interaction with Orai1. A stronger interaction between SOAR and Orai1 results in enhanced calcium influx. In addition, these results show that the I364 mutation recapitulates the effect of cholesterol removal in the SOAR-Orai1 complex.

### SOAR associates to the plasma membrane via cholesterol interactions

To explore in greater detail the molecular mechanisms responsible for protein-membrane interactions with SOAR, we carried out 400 ns molecular dynamics (MD) simulations of the SOAR fragment embedded in a single component POPC (1-palmitoyl-2-oleoyl-*sn*-glycero-3-phosphocholine) lipid bilayer and in a POPC:cholesterol (6:4 mol) lipid bilayer. The all atom simulations of the protein, solvent and phospholipid bilayer provide a very detailed atomistic picture of the specific protein-cholesterol interactions that determine the experimentally observed over-stabilization of the protein-membrane interaction in presence of cholesterol.

**Figure 4:**
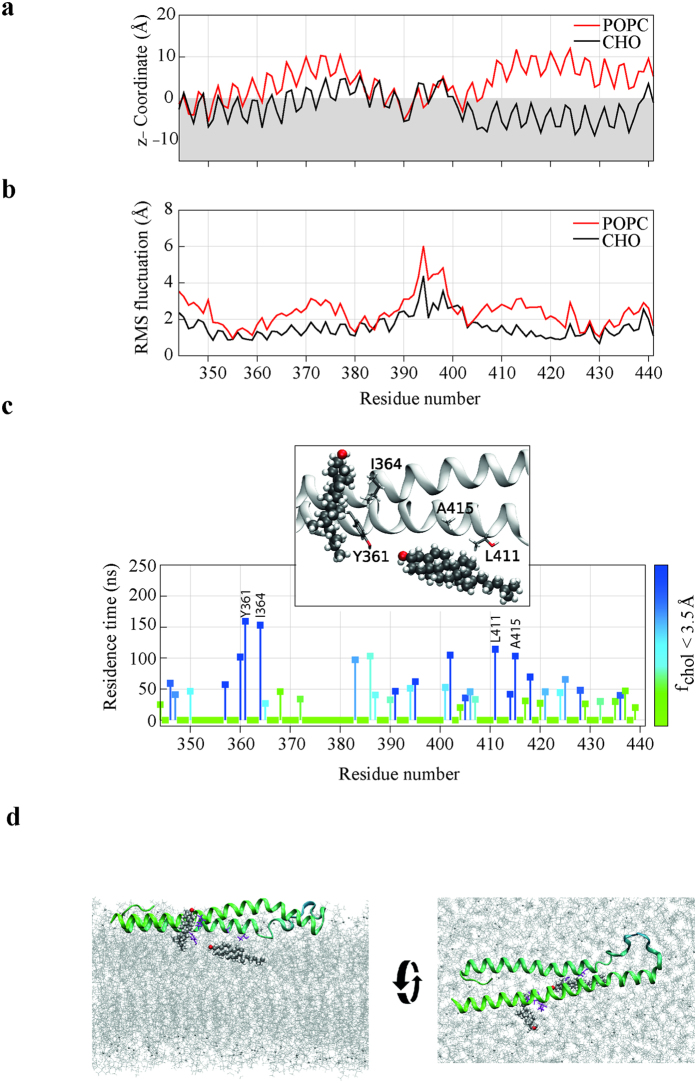
SOAR is positioned in a juxtamembranal plane to interact with cholesterol. (**a**) Lipid accessibility of each residue of the SOAR fragment derived from the MD simulation results. The lines depict the z-Coordinate insertion of each residue relative to the POPC head group region in a single component POPC lipid bilayer (Red line) and in a POPC:cholesterol (6:4 mole) lipid bilayer (Black line). (**b**) The Average root mean square fluctuations (RMSF) of each amino acid atoms as a function of residue number calculated from the SOAR fragment MD simulations in a single component POPC bilayer (Red line) and in a POPC:cholesterol (6:4 mol) bilayer (Black line). (**c**) The residence time (ns) for cholesterol molecules at 3.5 Å of each amino acid obtained by the MD simulations of the SOAR fragment in presence of cholesterol. The color scale represents the fraction of time that any cholesterol molecule was found within 3.5 Å of each residue. The longer residence time is presented as dark blue, mine while light green represent shorter residence time. Inset, is presented the avidity of SOAR residues to interact with cholesterol. (**d**) MD snapshots of the SOAR fragment (green) structures interacting with the POPC bilayer (gray lines), and cholesterol molecules from the MD simulation of the SOAR fragment in presence of cholesterol.

In the MD simulations without cholesterol, the SOAR fragment quickly drifts away from the hydrophobic POPC bilayer and none of the residues get deep inserted into the hydrophobic section of the bilayer (Fig. 4a). When the lipid bilayer contains cholesterol (6:4 mole), the SOAR fragment stabilizes paralleled to the bilayer and protrude into the phosphate group and interact tightly with the hydrophobic region of the lipid bilayer (Fig. 4a,d). The root mean square fluctuations (RMSF) reflect that mobility of the SOAR fragment is greatly reduced when the membrane contains cholesterol, compared to the mobility of each residue when the lipid bilayer does not contain cholesterol molecules (Fig. 4b).

The MD simulation results confirm that cholesterol in the lipid bilayer strongly influence the SOAR binding to the hydrophobic membrane. The residence time for cholesterol at each protein amino acid obtained by the MD simulations define two specific interactions between cholesterol molecules and the SOAR fragment: the hydrophobic region of I364 and Y361 forms the first strongest cholesterol-protein interaction and the second cholesterol-protein interaction was found among the cholesterol rings and a hydrophobic plane formed between L411 and A415 plus a polar interaction of the cholesterol hydroxyl and Y361 (Fig. 4c).

All the simulations recapitulate the experimental observations, indicating that cholesterol associates to STIM1 via I364 within the SOAR region. This interaction reduces the mobility of SOAR stabilizing this region paralleled to the plasma membrane, while enhancing the binding of STIM1 to Orai1 channel.

### STIM1 I364A role in Orai1 activation

**Figure 5:**
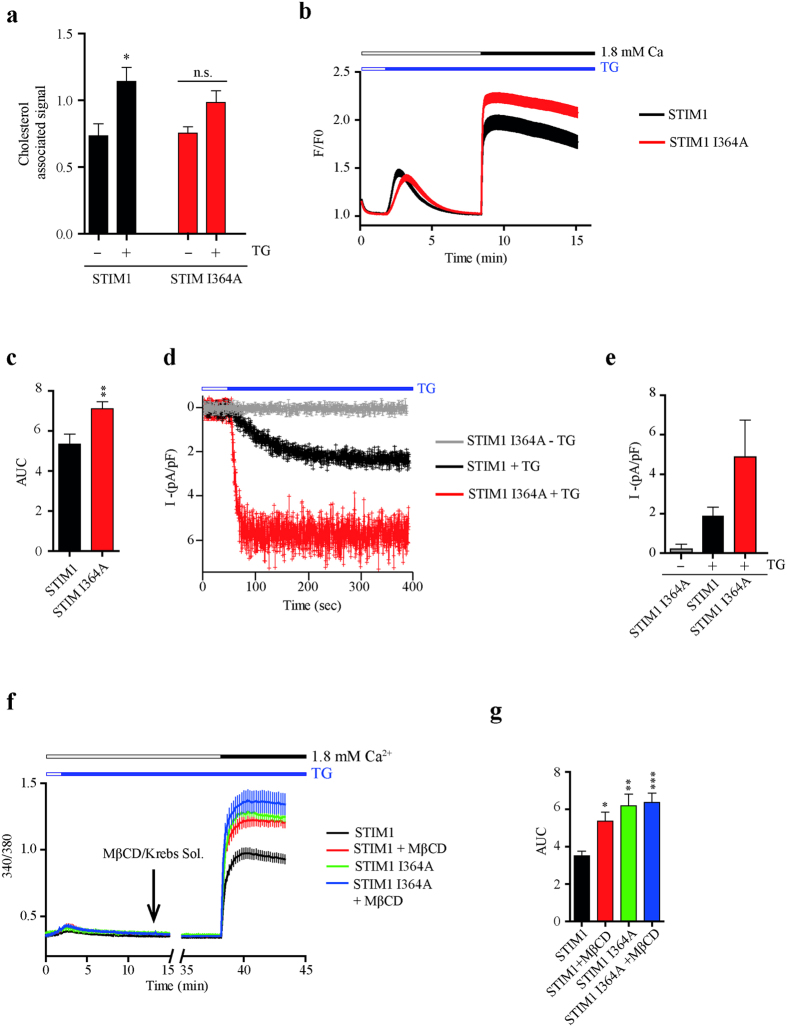
Isoleucine 364 mutant recapitulates cholesterol poor phenotype in STIM1. (**a**) Endogenous cholesterol associated to immunoprecipitates of STIM1 and STIM1 I364A. Bar graph show cholesterol signal normalized with CFP fluorescence in each immunoprecipitate. Data summarize results from at least 6 independent experiments. (**b**) Average calcium measurements of cells expressing Orai1 and STIM1 WT (n = 38 cells) or mutant I364A (n = 49 cells). Black and Red line respectively. TG (1 μM) was used to deplete the stores before add back calcium. (**c**) Summary graph bars of area under the curve (AUC) from calcium influx obtained from b. (**d**) Time courses for whole-cell inward current activation in cells overexpressing STIM1 and Orai1 (black trace) and cells overexpressing STIM1 I364A and Orai1 (Red trace). Upper blue rectangle shows the application of TG. Gray line depicts cells expressing STIM1 I364A and Orai1 without TG stimulus. (**e**) Bar graph summarizes current density of traces like in panel **d**. Data represent mean ± standard deviation from at least 15 cells from 3 independent transfections. (**f**) Average population calcium measurements from at least 4 independent experiments with a long protocol. STIM1/Orai1 complex formation was triggered by store depletion with TG. STIM1/Orai1 complex was allowed to form for 11 min before the addition of MβCD or calcium-free Krebs solution as control. Extracelullar calcium was added after 25 min of incubation with MβCD. (**g**) Bar graph summarizes calcium influx form experiments in f. All experiments were performed co-transfected with Orai1. Bars represent mean ± s.e.m. **p < 0.01 by two-tailed Student’s t-test in panel **c**. *p < 0.05, **p < 0.01, ***p < 0.0001 by one-way ANOVA with Bonferroni post-test analysis in g comparing with STIM1 as control.

To determine if effects of I364 mutation could be observed using the full length STIM1, we proceeded to evaluate the role of this single point mutation in our experiments using the full length STIM1 molecule. First, endogenous cholesterol associated to immunoprecipitate assays showed that STIM1 I364A mutation produced a non-significant increase of cholesterol associated when cells were treated with TG (Fig. 5a). Then, we used the classical protocol to study calcium entry mediated by SOCE. In agreement to the effect observed with SOAR I364A (Fig. 3c), STIM1 I364A showed a large increment in cytoplasmic calcium compared to cells transfected with STIM1 WT (Fig. 5b). The area under the curve shows significant differences between STIM1 WT and the mutant STIM1 I364A (Fig. 5c).

These results were corroborated by whole-cell current in cells expressing STIM1 WT or STIM1 I364A. Expression of STIM1 I364A mutant resulted in increased currents in comparison to cells expressing STIM1 WT (Fig. 5d,e).

To evaluate the effect of cholesterol depletion with STIM1 I364A, we conducted calcium measurements in cells already stimulated with TG (once the STIM1-Orai1 complex has been assembled). Using this protocol, calcium influx through STIM1-Orai1 complex can be evaluated in cholesterol poor conditions without affecting STIM1 puncta formation. The results obtained with full length STIM1 I364A recapitulated the observations with SOAR I364A (Fig. 3). Calcium influx was similar in cells expressing STIM1 wild type after MβCD treatment compared to cells expressing STIM1 I364A without MβCD (Fig. 5f,g). Thus, these results indicate that I364 is an important amino acid implicated in cholesterol regulation over SOAR and in the full length STIM1.

### Cholesterol regulation is coordinated by Orai1 and STIM1

**Figure 6:**
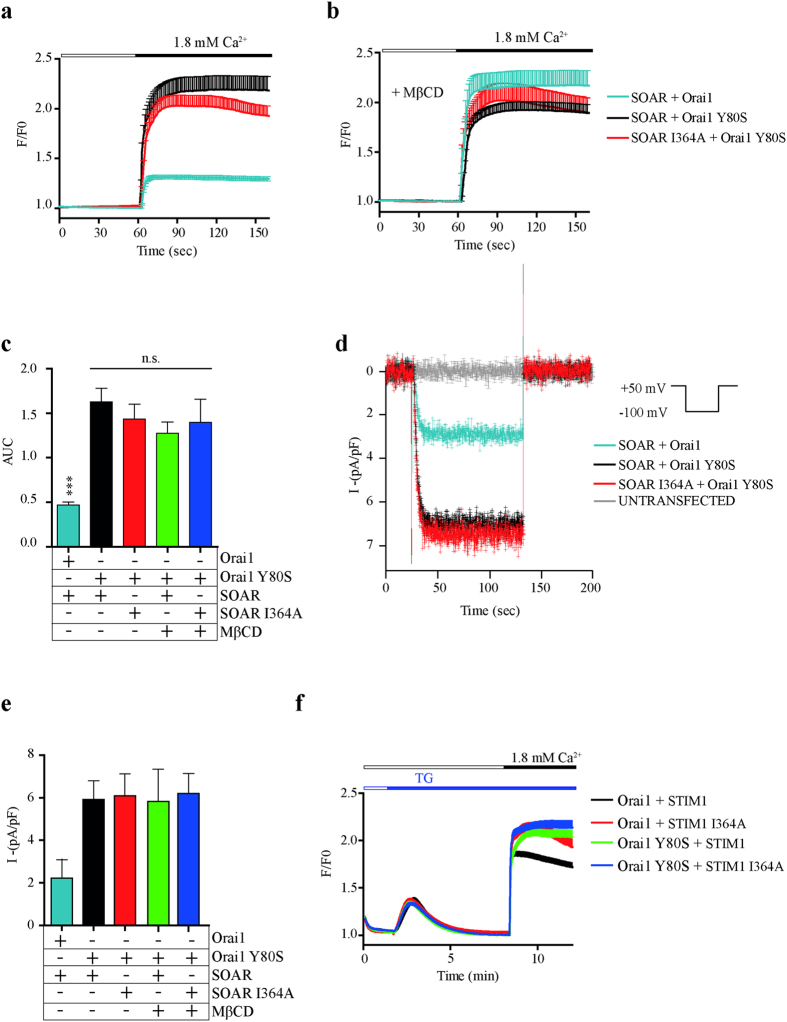
Cholesterol regulation of Orai1 is the same mechanism involved SOAR. (**a**) Average cytosolic calcium measurements of cells stimulated with 1.8 mM of extracellular calcium. Light blue line shows cells transfected with SOAR and Orai1 as control (n = 35). Black and red traces show cells transfected with SOAR and Orai1 Y80S (n = 27) and SOAR I364A with Orai1 Y80S (n = 24) respectively. For clarity, error bars are shown only from above of the mean. (**b**) Same conditions as in panel **a** plus MβCD treatment. Black and red traces show cells transfected with SOAR and Orai1 Y80S (n = 17) and SOAR I364A with Orai1 Y80S (n = 12) respectively (**c)** Quantification of area under the curve (AUC) from panel **a**,**b**. (**d**) Whole-cell patch-clamp recordings of cells transfected with Orai1 and Orai1 Y80S in combination with SOAR I364A and wild type. Black and red traces represent cells expressing SOAR and SOAR I364A with Orai1 Y80S respectively. Gray trace shows untrasfected cells. Light blue line shows cells transfected with SOAR and Orai1 as control. The protocol to obtain these traces is shown at top right. Data represent mean ± standard deviation from at least 15 cells from 3 independent transfections. (**e**) Bar graph summarizes current density of traces like in panel **d**. (**f**) Average calcium measurements of cells expressing STIM1 WT and mutant I364A in combination with Orai1 WT or Y80S. Black and Red line show cells expressing Orai1 WT with STIM1 (n = 24) and STIM1 I364A (n = 17) respectively. Green and Blue line show cells expressing Orai1 Y80S with STIM1 (n = 16) or STIM1 I364A (n = 22) respectively. TG (1 μM) was used to deplete the stores before add back calcium.

It has been published recently that cholesterol depletion upregulates SOCE by a CB domain present on the N-terminal region of Orai1^32^. Giving such result, we explored the effect of the Orai1 Y80S (a mutant of Orai1 with the CB domain abrogated) in combination with the SOAR and STIM1 I364A mutants. First, cells expressing Orai1 Y80S and SOAR wild type in standard cholesterol levels showed a significantly increased calcium influx compared to cells expressing Orai1 and SOAR wild types. Interestingly, SOAR I364A co-expressed with Orai1 Y80S presented almost the same increment than SOAR wild type with Orai1 Y80S (Fig. 6a). In addition, cholesterol depletion does not increased the calcium response of both, SOAR and SOAR I364A in combination with Orai1 Y80S (Fig. 6b). Results are summarized in Fig. 6c as area under de curve, showing no significant differences between SOAR and SOAR I364A with Orai1 Y80S, with or without MβCD. These results were corroborated by whole-cell current measurements in which cells expressing Orai1 Y80S also showed an increased current density when was co-expressed with SOAR wild type or SOAR I364A (Fig. 6d). Cholesterol reduction does not modify the amount of current density when Orai Y80S was co-expressed with SOAR wild type or SOAR I364A (Fig. 6e). These results indicate that abrogating the cholesterol-binding domain (CB) from either Orai or STIM1 is sufficient to enhance calcium influx and Orai currents.

Next we tested Orai1 Y80S in combination with the full-length STIM1 wild type or I364A mutant. The TG-evoked calcium entry of cells co-expressing Orai1 Y80S and STIM1 or STIM1 I364A was enhanced in comparison with cells expressing STIM1 and Orai1 wild types. Calcium influx was increased at comparative levels to cells expressing Orai1 and STIM1 I364A (Fig. 6d).

All together, these results strongly suggest that the CB domain of both, Orai1 and STIM1 coordinate the same mechanism mediated by cholesterol. And abrogation of either CB domain alone is sufficient to mimic the effect of cholesterol removal from the PM.

## Discussion

Several studies have shown that cholesterol modulates SOCE and influence STIM1 functionality^18^,^19^,^33^. However, the present study provides the first evidence of the presence of a CB domain in STIM1, positioned within SOAR. Previous studies about functional regulation of SOCE by cholesterol remarked the strong effect of inhibition of thapsigargin-evoked calcium entry after removing cholesterol, all mediated by abolition of STIM1 puncta formation that prevented Orai1 activation^18^,^20^,^22^. Notwithstanding, during the submission of this work, Romanin’s group published the opposite effect on SOCE when cholesterol is removed. This effect is mediated by cholesterol interacting with the extended transmembrane Orai1 N-terminal domain (ETON)^32^. The present study and that published by Romanin’s group indicate that cholesterol regulates both, STIM1 and Orai1 by interaction with two CB domains that act in a coordinated fashion, giving the same result: enhanced SOCE. Here we evaluated the role of cholesterol once the STIM1/Orai1 complex has been formed. Removing cholesterol under these conditions causes a massive increase in calcium entry. This increase is in agreement to the observations made by Galan *et. al*., who showed that depletion of cholesterol before TG addition inhibits calcium entry, but on the other hand, TG administration before removal of cholesterol enhances calcium influx^22^.

Exploring the molecular mechanism that promotes an increased calcium entry when removing cholesterol after the STIM1/Orai1 complex has been assembled, we found a single amino acid responsible for interacting with cholesterol within SOAR. SOAR association to cholesterol reduces the interaction between STIM1 and Orai1, functioning as a negative regulator of SOCE. This region interacts with cholesterol only after depletion of the ER when STIM1 unfolds to expose the SOAR domain. This region is not involved in the inhibition of STIM1 puncta observed when removing cholesterol prior to STIM1-Orai1 complex formation. Furthermore, STIM1 I364A mutant behaved similarly to wild type STIM1 when cholesterol was removed prior to SOCE activation with TG.

Our results suggest that STIM1 is actively interacting with cholesterol after calcium stores depletion via its CB domain. Immunoprecipitated STIM1 contains more endogenous cholesterol associated after calcium store depletion.

Peptide arrays pinpoint the sequence interacting with cholesterol, which maps inside SOAR. Although the CB consensus sequence L/V-X_(1–5)_-Y-X_(1–5)_-R/K results in a large number of potential cholesterol-binding regions, other criteria that increases the probability to identify a functional CB domain include the need for the region to be immersed in transmembrane or juxtamembranal regions. Also important to consider is the possibility that the protein containing a CB domain may have been identified as a constituent of cholesterol enriched domains. Finally, CB motifs fall in secondary structure of α-helixes^34^,^35^. STIM1 fulfills all these criteria. In spite of the fact that isoleucine is not part of traditional CB domains, our peptide array, molecular dynamics and functional studies indicate that this amino acid is essential for STIM1 cholesterol interaction. On the other hand tyrosine Y361, which is part of consensus CB domains, did not show any phenotype in cholesterol-depleted cells. Nevertheless, we do not discard that other residues within STIM1 may be involved in cholesterol interactions.

SOAR (or CAD) fragment has shown to be sufficient to promote clustering of Orai1 channels and activation of calcium influx^3^. Our studies indicate that the positioning of SOAR in closed proximity to the PM occurs via the interaction of I364 with cholesterol (Figs 3 and 4). Such positioning may be relevant for the concomitant interaction between SOAR and Orai1. Our measurements indicate that SOAR is located within 3.2 ± 0.17 nm from the PM, in agreement with a previous study reporting a distance of 4–6 nm to accommodate full-length STIM1 molecules in ER-PM junctions^36^.

Our molecular dynamic simulations show that the whole cholesterol molecule interacts with SOAR, stabilizing the protein when cholesterol is present in the plasma membrane. Experimental results match the molecular dynamic simulations, indicating that the distance between SOAR and the PM increase in cholesterol-depleted conditions, which may facilitate the subsequent association to Orai1.

A perturbation on Orai1 structure induced by the lack of cholesterol (as concluded in the study by Romanin’s group) may facilitate the association of SOAR to the channel^32^. In addition, the instability of SOAR in cholesterol depleted membranes could produce a better anchoring to the N-terminal of Orai1. On the light of these results it is necessarily to conduct further studies to solve the molecular role of cholesterol in STIM1 and Orai1 as well as the association order of STIM1-Orai1-cholesterol on first steps of activation of SOCE.

Heterogeneous lipid distribution in the PM and its implications on STIM1-Orai1 complex are recently emerging. PIP2-rich domains are recognized to facilitate slow Ca^2+^-dependent inactivation (SCDI) of Orai1 in a mechanism dependent of STIM1-SARAF interaction^11^. In addition, there is strong evidence showing poor and enriched cholesterol microdomains on the PM^37^,^38^,^39^,^40^.

SOCE complex established in microdomains enriched in cholesterol are especially important for functionality in immune cells^41^, where polarized distribution of STIM1 at the immunological synapse has been shown to be a primary event during NFAT activation^42^,^43^. Cholesterol is very abundant at the immunological synapse^44^,^45^. There are also reports showing a critical specific Ca^2+^ pattern to trigger NFAT nuclear translocation 46,47. We suggest that distinct cholesterol microdomains could be regulating Orai1 once activated by STIM1.

## Methods

### Cell culture and transfection

HEK293 cells were grown in DMEM supplemented with 10% (V/V) fetal bovine serum, 50 μg/ml penicillin, 50 μg/ml streptomycin and maintained at 37 °C in a humidified atmosphere of 5% CO_2_ and 95% air. For imaging experiments, one day prior to transfection with the plasmid of choice, the cells were plated onto 25 mm circular coverslips. Plasmids of EGFP-SOAR, EGFP-SOAR L/Q, YFP-STIM1 and mCherry-Orai1 were a generous gift from S. Muallem. STIM1-CFP and STIM1-YFP were a generous gift of M. Prakriya. Myc-Orai1 Y80S was a generous gift of Richard Lewis and YFP-Orai1 and Orai1-myc were purchased from Addgene. All mutations in STIM1 and SOAR were generated using the QuikChange site-directed mutagenesis kit (Stratagene). Cells were transient transfected with lipofectamine 2000 (Invitrogen) according to manufacturer’s instructions using 1 μg of total cDNA for a 25 mm coverslip. Cells were analyzed 1 day after transfection.

### Cholesterol treatments

For all experiments, except those from Fig. 5f cholesterol extraction was performed by using 7.5 mM of MβCD dissolved in Krebs solution (119 mM NaCl, 2.5 mM KCl, 1 mM NaH_2_PO_4_, 1.3 mM MgCl_2_, 20 mM HEPES, 11 mM glucose and adjusted to 7.4 pH) for 1.5 hours. Prior to MβCD cell culture was rinsed twice with krebs to remove residues of serum. In Fig. 5f 10 mM of MβCD was used. For filipin treatments we incubated cells with Filipin III at 1 μg/ml dissolved in calcium-free Krebs solution for 1 hour at 37 °C. Filipin III was used from a stock of 1 mg/ml dissolved in DMSO. To determine the cholesterol amounts of cells treated with 7.5 mM of MβCD and untreated cells. Cells were growth in 96 wells plates and analyzed in a Synergy Mx microplate reader. We used a standard Amplex® Red cholesterol assay kit (Molecular Probes) according to manufacturer’s instructions. Amplex® Red was excited at 540 nm and emission was collected at 590 nm with a 30 nm bandpass. In the same way, cell viability was measured using Live/Dead® viability/cytotoxicity kit (Molecular Probes). Calcein (live cells) was excited at 494 nm and emission was collected at 517 nm using a 9 nm bandpass. Ethidium homodimer-1 (dead cells) was measured using 528 and 617 nm for excitation and emission, respectively with a 30 nm bandpass.

For experiments to evaluate the specificity of MβCD, cells were incubated for 1.5 hours with Krebs solution saturated with cholesterol. Krebs solution was prepared by using equimolar ratios of MβCD:Cholesterol at 7.5 mM. MβCD:Cholesterol complex was dissolved by vortexing and sonication for 5 min and filtered through a 0.2 μm syringe filter to remove undissolved cholesterol crystals.

### Co-immunoprecipitation

Cells grown in 60 mm dishes and transfected with SOAR and Orai1-myc were lysed using 300 μl of PBS buffer containing 5% Triton X-100 and proteases inhibitor cocktail (Roche). The cells extracts were incubated for 2 hours at 4 °C and centrifuged at 13600 rpm for 20 minutes, then the supernatant was recovered. Immunoprecipitation was conducted incubating cell extracts with myc antibody (2.5 μg) for 2 hours and then with 30% of volume with protein A/G Sepharose beads for 3 hours at 4 °C. Beads were washed four times with PBS buffer without Triton X-100. Proteins were eluted from the beads with Laemmli buffer at 90 °C for 5 min. For Western Blot analysis, proteins were resolved using 12% SDS-polyacrylamide gels and transferred to a nitrocellulose membrane. The blot was stripped and re-tested when needed. The stripping solution containing glycine 0.2 M, SDS 0.1%, Tween 20 at 1% and pH adjusted to 2.2. The blot was incubated with stripping solution for 20 min at 80 °C and 5 min with fresh stripping solution before to testing again.

### Peptide arrays

To screen important residues in cholesterol interact within SOAR we used a modified version of lg-TIRFM (TIRF Labs, Inc., Cary, NC.) as previously described^48^,^49^. Briefly, 30 length aminoacids covering from 350 to 380 of STIM1 were synthetized. All peptides were purchased from JPT Peptide Technologies (Berlin, Germany). Peptide spots were printed manually by means of a TIRF MicroArrayer (TIRF Labs, Cary, NC.) on conventional microarray slides coated with poly-L-lysine (Sigma, St. Louis, MO.). Lg-TIRFM-1000 system in combination with an iXon electron-multiplying charge-coupled device (EMCCD) camera (Andor Technology, South Windsor, CT.) were used to monitor fluorescence of peptide arrays incubated with 10 μM of Dehydroergosterol (DHE). Image analysis was conducted with Igor pro software (wavemetrics, Portland, OR.).

### Calcium measurements

Calcium imaging was performed in 25 mm coverslip with cells transfected with the plasmid of choice indicated in the figure legends. Cells were loaded with Fluo4-AM (Molecular Probes) at 2 μM final concentration in Krebs solution and incubated by 30 min at 25 °C. Calcium-free Krebs solution containg: 119 mM NaCl, 2.5 mM KCl, 1 mM NaH_2_PO_4_, 1.3 mM MgCl_2_, 20 mM HEPES, 11 mM glucose, 500 μM EGTA and adjusted to 7.4 pH. To deplete calcium stores 1 μM of thapsigargin (TG) was used followed by addition of 1.8 mM of calcium. Fluorescent registration was conducted in a wide-field inverted IX81 Olympus® microscope equipped with 40 × 1.30 NA objective, MT-20 illumination system, 484/25 excitation filter, 520 nm/40 bandpass emission filter with an EMCCD camera iXon-897 (Andor Technology South Windsor, CT, USA). Images were analyzed with Olympus Cell^R software.

Long protocol to estimate calcium entry after MβCD treatment was performed in 35 mm dishes with cells transfected with the plasmid of choice. Each dish containing cells was loaded with FURA-2 AM (Molecular Probes) at 2 μM final concentration in Krebs solution and incubated by 30 min at 25 °C. Calcium-free Krebs solution (119 mM NaCl, 2.5 mM KCl, 1 mM NaH_2_PO_4_, 1.3 mM MgCl_2_, 20 mM HEPES, 11 mM glucose, 1 mM EGTA and adjusted to 7.4 pH). 1 μM of TG was used to empty the stores for 11 min, followed by addition of 10 mM of MβCD or calcium-free Krebs solution as control. MβCD was incubated for 25 min, followed by the addition of 1.8 mM of calcium. Fluorescence records were performed through an Aminco-Bowman luminescence spectrometer (Thermo Electron, Madison, WI). Dual excitation wavelength was selected at 340 nm and 380 nm and emission was collected at 510 nm. Fluorescence sampling was acquired each 10 seconds.

### Cholesterol co-immunoprecipitation assay

To determine the presence of endogenous cholesterol in the protein of interest, we performed a cholesterol associated assay as previously described^25^. The following modifications were conducted: transfected cells were lysed with a buffer containing: 50 mM Tris-HCl, 150 mM NaCl, 50 mM NaF, 1 mM dithiothreitol, 0.5 mM PMSF, 50 mM sodium pyrophosphate, 10 mM sodium vanadate, 0.25% of sodium deoxicholate, 0.1% of Nonidet P40, 0.5% of Triton X-100, 0.1% of Digitonin, proteases inhibitor cocktail (Roche) and adjusted to 7.4 pH. Cell extracts were incubated with protein A/G Sepharose beads and GFP polyclonal antibody (Clontech) overnight at 4 °C. Beads were washed four times with the same lysis buffer. Protein was eluted from the beads with glycine 0.2 M at 2.5 pH. Then, elution was neutralized with a buffer containing Tris-HCl 20 mM, NaCl 137 mM and EDTA 2 mM at 8.0 pH. Finally, we measured cholesterol concentration in the solution with immunoprecipitated protein using Amplex® Red cholesterol assay kit (Molecular Probes). Reactions were measured in a Synergy Mx microplate reader. Cholesterol concentration was normalized to YFP or CFP fluorescence intensity in the same reaction. YFP was measured at 510/9 nm and 535/9 nm bandpass for excitation and emission respectively. CFP was measured at 405/9 and 485/9 bandpass for excitation and emission respectively. Data analysis was conducted through the ratio of Amplex red cholesterol signal divided by YFP or CFP fluorescence in each sample.

### Electrophysiology studies

HEK293 cells expressing the different constructs described in the figure legends were placed on coverslips coated with poly-lysine (Sigma). Cells were studied between 48–52 hours post-transfection. Coverslips were mounted on an open perfusion chamber (TIRF Labs).

The patch clamp amplifier used for whole-cell recordings was the EPC-9 (Heka Electronik, Germany). The patch clamp pipettes were prepared from Corning 7052 glass and had a resistance of 1–5 MΩ when filled with the pipette solution (see below). An Ag/AgCl electrode was utilized to attain electric continuity and was connected to the bath solution (see below) via a KCl agar bridge. TG was applied using a multibarrel perfusion system driven by gravity.

The pipette solution contained: Cesium aspartate 120 mM, EGTA 5 mM, HEPES 10 mM, MgCl_2_ 2 mM and NaCl 8 mM. pH to 7.2 adjusted with CsOH. The bath solution contained: NaCl 120 mM, Tetraethylammonium chloride (TEA-Cl) 10 mM, CaCl_2_ 10 mM, MgCl_2_ 2 mM, Glucose 30 mM and HEPES 10 mM. pH to 7.2 adjusted with NaOH. Osmolarity of both solutions was adjusted to 320 mosM with mannitol (Sigma).

For experiments using SOAR WT and mutant, cell membrane potential was maintained at +50 mV and a 100 second pulse to −100 mV was conducted to evaluate inward currents trough Orai1 channels. For experiments using full length STIM1 and mutant, membrane potential was maintained at −100 mV throughout the experiment and whole-cell currents were invoked by 1 μM TG application to the bath solution.

### Confocal imaging

All FRET experiments were performed in an Olympus® FV1000 spectral confocal microscope. The FRET efficiency between mCherry-Orai1 (acceptor) and GFP-SOAR (donor) was monitored by collecting fluorescence increments of GFP before and after photobleaching of mCherry. Transfected cells were excited using a 488 laser line for GFP and a 543 for mCherry. Emission was collected at 500/530 and 555/655 for GFP and mCherry channels, respectively. Images were taken each second, 5 images prior to photobleaching pulse, which lasted 10 seconds at 80% power of 543-laser line in a region of interest (ROI). Finally 10 images were taken to monitor fluorescence levels of each fluorophore. To eliminate fluorescence variations overtime, fluorescence fluctuations in non-photobleaching areas were subtracted from uncorrected FRET data (5). FRET efficiency was calculated with the formula *E*_*FRET*_ = *100*(*1* − *D*_*pre*_*/D*_*post*_), where *D*_*pre*_ and *D*_*post*_ is GFP fluorescence intensity before and after the photobleaching protocol, respectively.

We measured distance between plasma membrane and GFP-SOAR as previously described^50^, dipicrylamine (DPA) was used as a quencher acceptor of GFP. Cells were transfected with both, mCherry-Orai1 and GFP-SOAR. Donor fluorescent levels of GFP were acquired taking 4 images before the addition of 20 μM of DPA and 10 images after DPA addition. The images were taken every 2.5 min. To increase the closeness of DPA to the inner layer of the plasma membrane the last 2 points of fluorescence intensity were measured following the addition of high K^+^ solution (NaCl 95 mM, KCl 40 mM, HEPES 17 mM and adjusted to 7.4 pH)^31^. Low laser power (<10%) was used to avoid photobleaching during these experiments.

FRET by DPA quenching was calculated by the formula *E* = *1* − (*I*_*DA*_*/I*_*D*_), where *I*_*D*_was fixed at 1.0 and *I*_*DA*_ was taking at the last point of the experiment, after addition of high K^+^ solution. Line scan was used to acquire GFP fluorescent signal, the maximum signal on mCherry-Orai1 was taking at the plasma membrane in coincidence with GFP signal. To estimate the distance (*r*) from GFP-SOAR to the plasma membrane, we used the formula *r* = *R*_*0*_ [(*1/E*) − *1]*^*1/6*^, where *R*_*0*_ has a value of 37 Å according to^31^.

### MD simulation methods

All-atom simulations were carried out with the CHARMM36^51^,^52^ force field models for the protein, membrane and solvent environments. The initial coordinates of the monomeric peptide were generated using the available coordinates of the SOAR fragment from Homo sapiens (3TEQ)^4^, the protein orientation to the membrane was assigned using the OPM database^53^ and embedded in the corresponding lipid bilayers and 0.15 KCl ion concentrations using the CHARMM-GUI membrane builder^54^,^55^. The final composition for the SOAR-POPC simulation consists of the protein fragment, 299 POPC lipids, 13,123 water molecules, 41 K^+^ and 44 Cl^−^ atoms. For the 40% cholesterol simulations the system consists of 144 cholesterol molecules, 216 POPC lipids 14,383 water molecules, 43 K^+^ and 46 Cl^−^ atoms.

The system was energy minimized using steepest descent method and equilibrated at 310 K using NVT and NPT ensembles in which water, lipids, ions and the protein were constrained using a harmonic potential. At the final equilibration step all restraints were removed.

For the 400 ns production runs periodic boundary conditions were employed, the short-range electrostatic interactions were cut-off at 1.2 nm and the full electrostatics were treated using PME algorithm^56^. Van der Waal interactions were evaluated using a cutoff switch from 10 to 12 nm. To control the temperature and pressure at 310 K and 1 atm the Nosé-Hoover thermostat with a coupling constant of 1.0 ps and the Parrinello-Rahman barostat with a coupling constant of 1 ps and a compressibility of 4.5 × 10^−5^ bar ^−1^ were employed using a semi-isotropic coupling for pressure control^57^. The linear constraint solver (LINCS) method^58^ was used to constrain all bond lengths, and a 2 fs integration step was used. The simulations were carried out using GROMACS (v5.0.5)^59^ and the analyses were performed using python and the MDAnalysis libraries^60^. All molecular images were created using VMD^61^.

### Data analysis

Data were analyzed by one-way ANOVA followed by Bonferroni multiple comparisons tests or by two-tailed Student’s t-test (GraphPad Prism, GraphPad Software Inc). Unless otherwise indicated data are presented as means ± s.e.m. with significance set at ***p < 0.0001, **p < 0.01 or *p < 0.05.

## Additional Information

**How to cite this article**: Pacheco, J. *et al*. A cholesterol-binding domain in STIM1 modulates STIM1-Orai1 physical and functional interactions. *Sci. Rep*. **6**, 29634; doi: 10.1038/srep29634 (2016).

## Supplementary Material

Supplementary Information
