# Supplementary material for: A cholesterol-binding domain in STIM1 modulates STIM1-Orai1 physical and functional interactions
Source: Sci Rep. 2016 Jul 27;6:29634. doi: 10.1038/srep29634 (PMC4962086; doi:10.1038/srep29634)

Supplementary Information

A cholesterol-binding domain in STIM1 modulates STIM1-Orai1 physical and functional interactions

Jonathan Pacheco[1], Laura Dominguez[2], A. Bohórquez-Hernández[1], Alexander Asanov[3] and Luis Vaca[1]:

[1]*Instituto de Fisiología Celular, Universidad Nacional Autónoma de México, Ciudad Universitaria, México, DF 04510, México*

[2]*Departamento de Fisicoquímica, Facultad de Química, Universidd Nacional Autónoma de México, Ciudad Universitaria, México DF 04510, México.*

[3] *TIRFLabs Inc. 106 Grendon Place. Cary, NC 27519*

Authors correspondence: Dr. Laura Dominguez: lauraok@gmail.com. A. Bohórquez-Hernández: abohorquez@email.ifc.unam.mx. Dr. Alexander Asanov: alex.asanov@tirf-labs.com. Jonathan Pacheco: epacheco@email.ifc.unam.mx

***Corresponding author**: Dr. Luis Vaca. lvaca@ifc.unam.mx*

Supplemental figures legends

FIGURE S1. MβCD or filipin treatment increases SOCE in cells expressing SOAR

A, Average calcium response of HEK293 cells expressing SOAR and Orai1. Control cells present standard cholesterol conditions (Black line) and cells depleted of cholesterol were treated with 1µg/ml of filipin (Red line). **B,** Summary graph bars of area under de curve (AUC) obtained after extracellular calcium addition of control (n=9 cells) and filipin treated cells (n=35). **C,** Representative calcium measurements from HEK293 cell expressing SOAR and Orai1. Control cells (Black line) and cholesterol depleted cells treated with 7.5 mM MβCD (Red line). Thapsigargin (TG, 1 µM) was used to deplete the stores before adding calcium back to the bathing (extracellular) solution. **D,** Summary graph bars of peaks from the first and second calcium addition in control (n=49 cells) and MβCD treated cells (n=28 cells). Bars represent mean ± s.e.m. *** p<0.0001 by two-tailed Student's t-test.

FIGURE S2. MβCD treatment inhibits endogenous SOCE

A, Representative calcium time course of untransfected HEK293 cells. Black line shows control cells in standard cholesterol conditions and red line shows cells treated with 7.5 mM of MβCD for 1.5 hours before TG application. **B,** Summary graph bars of area under de curve (AUC) from calcium addition of experiments in A. Black bar represent untreated cells (n=15) and red bar shows cells treated with MβCD (n=27). Bars show mean ± s.e.m. *** p<0.0001 by two-tailed Student's t-test.

FIGURE S3. MβCD treatment removes cholesterol without affecting cell viability

A, Percentage of cholesterol extraction when cells were treated with MβCD. Black bar represent cells untreated and red bar show cells incubated 1.5 hours with 7.5

mM of MβCD. **B,** Cell viability after MβCD treatment. Black bar represent cells untreated and red bar shows cells after treatment with MβCD. Bars show mean ± s.e.m.

FIGURE S4. SOAR I364A mutant effect is not affected by removing cholesterol with filipin.

A, Average constitutive calcium response of HEK293 cells expressing SOAR I364A and Orai1. Cells without treatment presented standard cholesterol conditions (green line) and cells depleted of cholesterol were treated with 1µg/ml filipin (Blue line). **B,** Summary graph bars of area under de curve (AUC) after calcium addition to the extracellular (bathing) solution in control (n=35 cells) and filipin treated cells (n=26).

SUPPLEMENTAL FIGURE 1

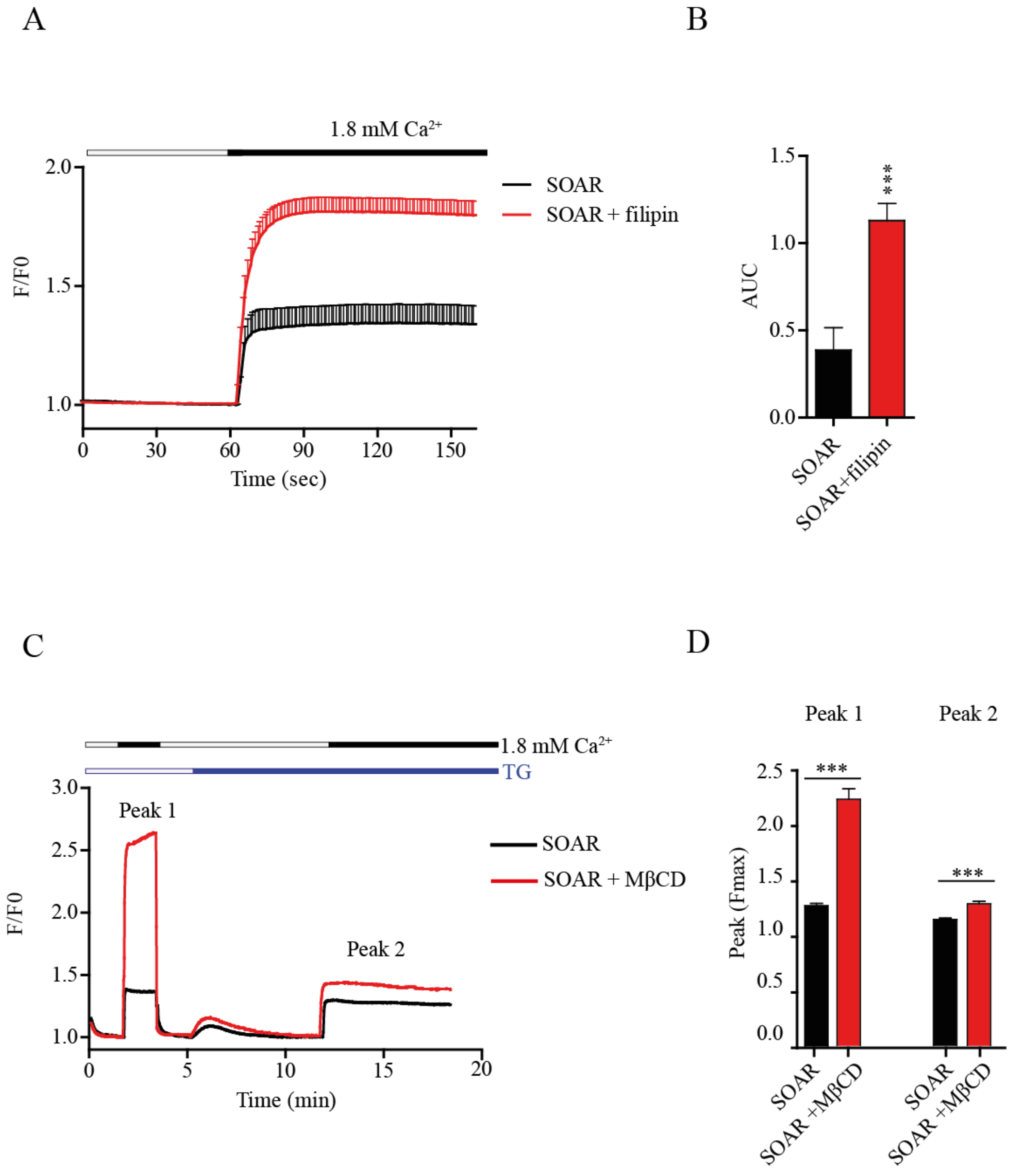

SUPPLEMENTAL FIGURE 2

A

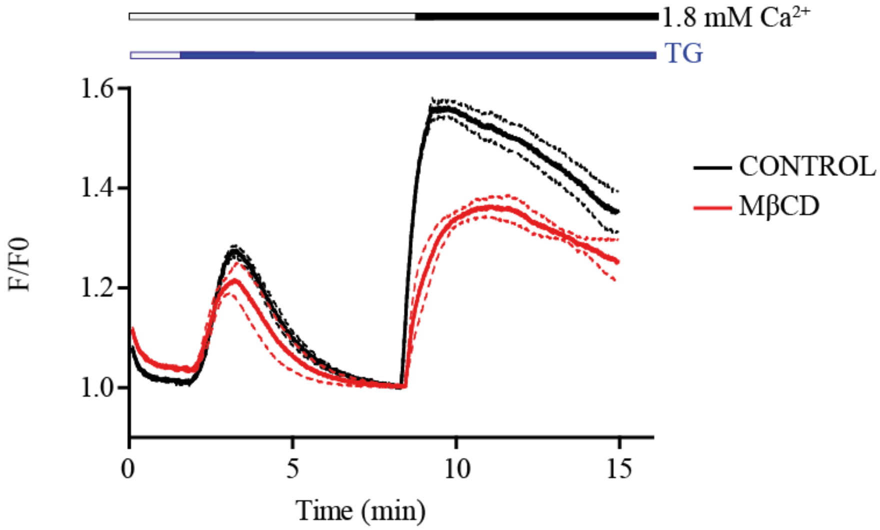

B

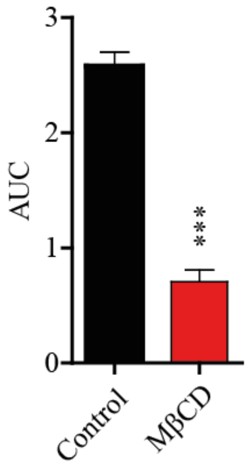

SUPPLEMENTAL FIGURE 3

A

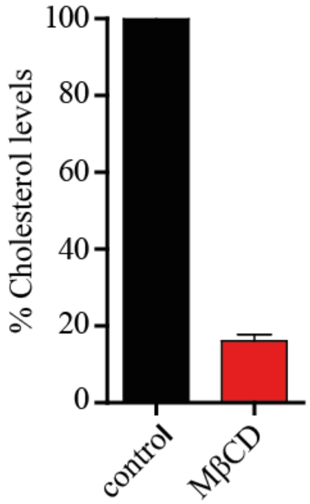

B

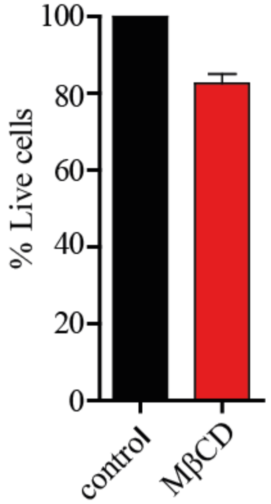

SUPPLEMENTAL FIGURE 4

A

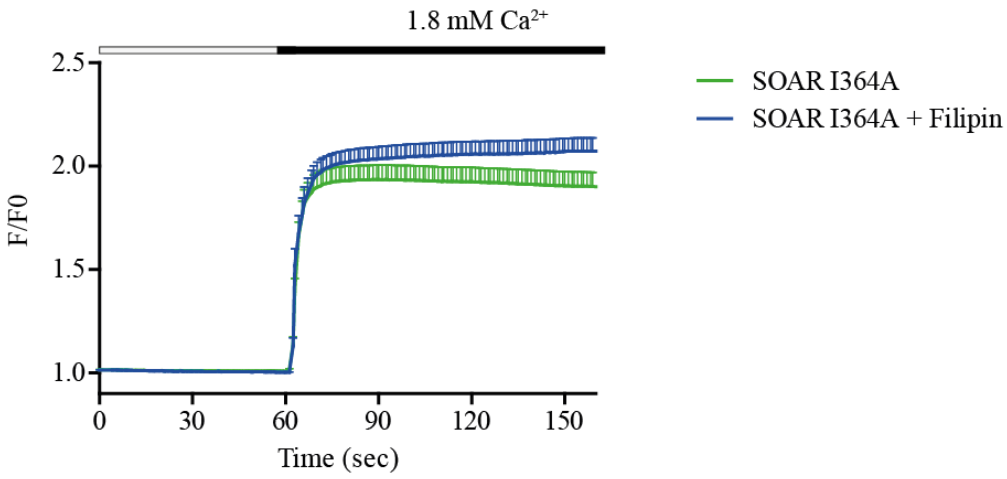

B

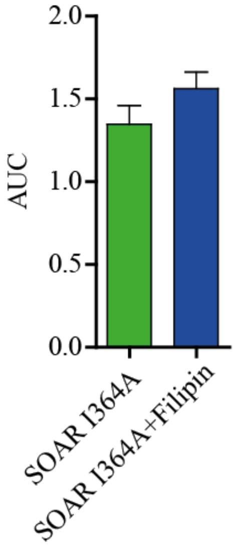

Supplement: Supplementary Information [file srep29634-s1.pdf]
